# Supplementary material for: Sequence and structure of protein binding sites in RNA impact biomolecular condensates
Source: Mol Biol Cell. 2026 Jun 1;37(6):ar53. doi: 10.1091/mbc.E26-02-0083 (PMC13537349; doi:10.1091/mbc.E26-02-0083)
Supplement: Supplementary file 1 [file mbc-37-ar53-s001.pdf]

Supplemental Materials

Molecular Biology of the Cell

Cole *et al*.

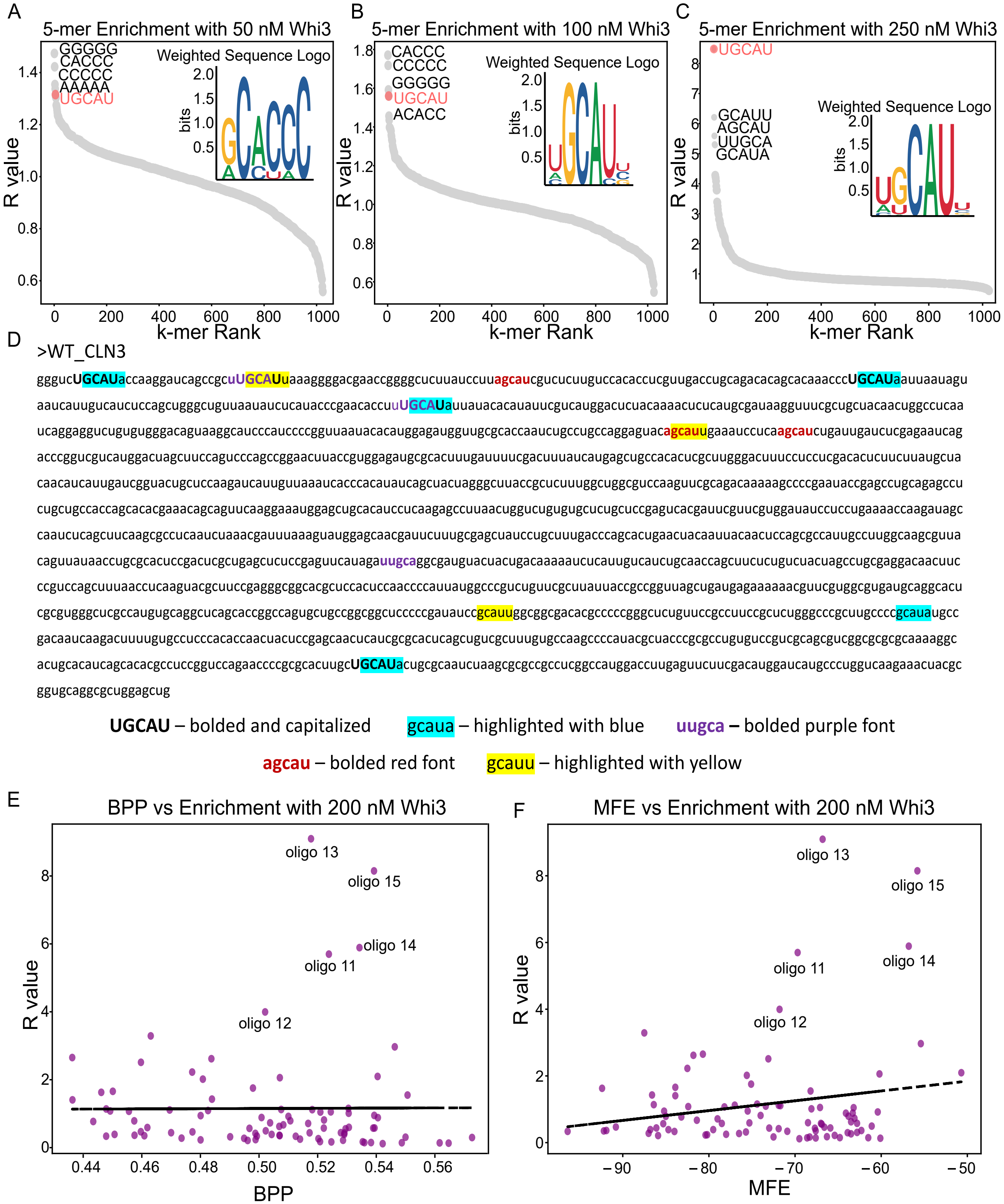
A
5-mer Enrichment with 50 nM Whi3
GGGGG
CACCC
CCCCC
AAAAA
UGCAU
Weighted Sequence Logo
bits
R value
k-mer Rank
B
5-mer Enrichment with 100 nM Whi3
CACCC
CCCCC
GGGGG
UGCAU
ACACC
Weighted Sequence Logo
bits
R value
k-mer Rank
C
5-mer Enrichment with 250 nM Whi3
UGCAU
GCAUU
AGCAU
UUGCA
GCAUA
Weighted Sequence Logo
bits
R value
k-mer Rank
D
>WT_CLN3
gggucUGCAUaccaaggaucagccgcuUGCAUuaaaggggacgaaccggggcucuuauccuuagcaucgucucuuguccacaccucguugaccugcagacacagcacaaacccUGCAUaauuaauagu
aaucauugucaucuccagcugggcuguuaauaucucauacccgaacaccuuUGCAUauuauacacauauucgucauggacucuacaaaacucucaugcgauaagguuucgcugcuacaacuggccucaa
ucaggaggucuguguggggacaguaaggcaucccaucccgguuaauacacauggagaugguugcgcaccaaucugccugccaggaguacagcauugaaauccucaagcaucugauugaucucgagaaucag
acccggucgucauggacuagcuuccagucccagccggaacuuaccguggagaugcgcacuuugauuuucgacuuuaucaugagcugccacacucgcuugggacuuuccuccucgacacucuucuuaugcua
caacaucauugaucgguacugcuccaagaucauuguuaaaucacccacauaucagcuacuagggcuuaccgcucuuuggcuggcguccaaguucgcagacaaaaagccccgaauaccgagccugcagagccu
cugcugccaccagcacacgaaacagcaguucaaggaaauggagcugcacauccucaagagccuuaacuggucugugugcucugcuccgagucacgauucguucguggauauccuccugaaaaccaagauagc
caaucucagcuucaagcgccucaaucuaaacgauuuaaaguauggagcaacgauucuuugcgagcuauccugcuuugacccagcacugaacuacaauuacaacuccagcgccauugccuuggcaagcguua
caguuauaaccugcgcacuccgacucgcugagcucuccgaguucauagauugcaggcgauguacuacugacaaaaaucucauugucaucugcaaccagcuucucugucuacuagccugcgaggacaacuuc
ccguccagcuuuaaccucaaguacgcuuccgagggcggcacgcuccacuccaaccccauuauggcccgucuguucgcuuauuaccgccgguuagcugaugagaaaaaacguucguggcgugaugcaggcacu
cgcgugggcucgccaugugcaggcucagcaccggccagugcugccggcggcucccccgauauccgcauuggcggcgacacgcccccgggcucuguuccgccuuccgcucugggcccgcuugccccgcauaugcc
gacaaucaagacuuuugugccucccacaccaacuacuccgagcaacucaucgcgcacucagcugucgcuuugugccaagccccauacgcuacccgcgccuguguccgucgcagcgucggcgcgcgcaaaaggc
acugcacaucagcacacgccuccgguccagaaccccgcgcacuugcUGCAUacugcgcaaucuaagcgcgccgccucggccauggaccuugaguucuucgacauggaucaugcccuggucaagaaacuacgc
ggugcaggcgcuggagcug
UGCAU – bolded and capitalized
gcaua – highlighted with blue
uugca – bolded purple font
agcau – bolded red font
gcauu – highlighted with yellow
E
BPP vs Enrichment with 200 nM Whi3
oligo 13
oligo 15
oligo 14
oligo 11
oligo 12
R value
BPP
F
MFE vs Enrichment with 200 nM Whi3
oligo 13
oligo 15
oligo 11
oligo 14
oligo 12
R value
MFE

A

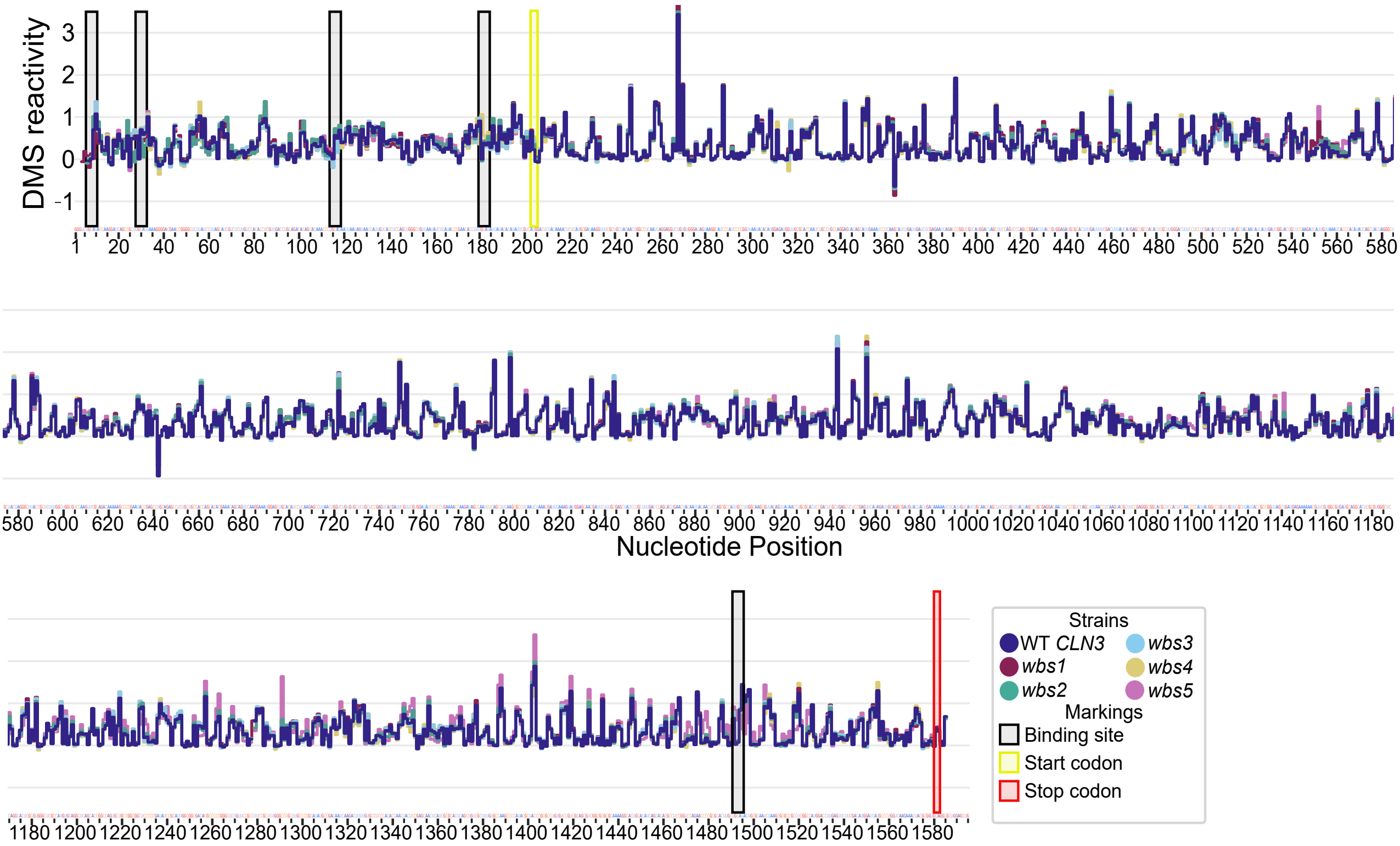
DMS reactivity
Nucleotide Position
Strains
WT CLN3
wbs1
wbs2
wbs3
wbs4
wbs5
Markings
Binding site
Start codon
Stop codon

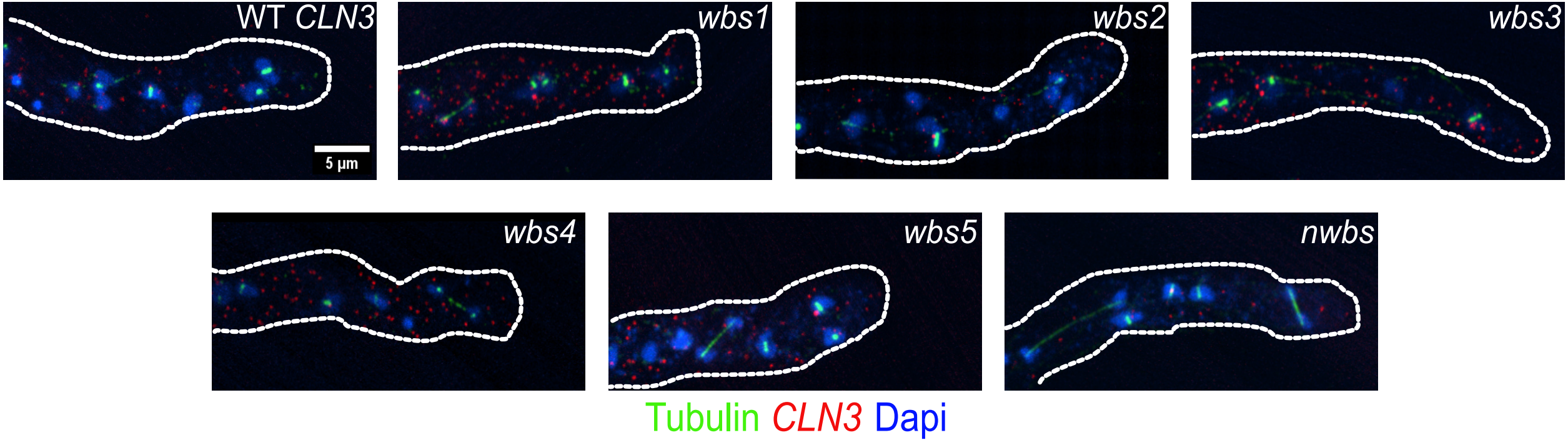
WT *CLN3*
5 µm
wbs1
wbs2
wbs3
wbs4
wbs5
nwbs
Tubulin *CLN3* Dapi

Supplemental Figure 1. Determining primary RNA interaction sites for Whi3. A) A graph showing which 5 nucleotide motifs were most enriched in the random oligo pool RBNS experiment at 50 nM Whi3, with UGCAU containing motifs highlighted in red. B) A graph showing which 5 nucleotide motifs were most enriched in the random oligo pool RBNS experiment at 100 nM Whi3. C) A graph showing which 5 nucleotide motifs were most enriched in the random oligo pool RBNS experiment at 250 nM Whi3. D) Pearson correlation of *CLN3* oligo base pair probability and R value score. Pearson's r = 0.01 p-value = 0.954 E) Pearson correlation of *CLN3* oligo minimum free energy (MFE) predictions and R value score (Gruber et al., 2008). Pearson's r = 0.19 p-value = 0.0764 F) Pearson correlation of *CLN3* oligo ensemble diversity (ED) predictions and R value score (Gruber et al., 2008). Pearson's r = -0.06 p-value = 0.588

Supplemental figure 2. Normalized DMS reactivity profile of all *CLN3* strains with binding sites highlighted with grey boxes, start codon highlighted in yellow box, and stop codon highlighted in red box.

Supplemental figure 3. Representative images of the IF FISH data for each mutant shown with tubulin IF in green, *CLN3* FISH in red, and Dapi staining in blue. The same brightness and contrast was used for all images. Scale bar is 5 microns.

RNA type	Minimum free energy (kcal/mol)	2X MFE (kcal/mol)	Co-fold dimerization prediction (kcal/mol)
WT	-604.85	-1209.7	-1230.62
wbs1	-601.86	-1203.72	-1225.38
wbs2	-604.68	-1209.36	-1230.28
wbs3	-604.39	-1208.78	-1228.6
wbs4	-605.64	-1211.28	-1230.74
wbs5	-605.28	-1210.56	-1230.02
nwbs	-527.75	-1055.5	-1183.98

Supplemental Table 1: Minimum free energy values predicted using Vienna RNA and predicted co-fold dimer minimum free energy at 25 °C and 150 mM salt concentration (Gruber et al., 2008).
